# Vicarious Learning from Human Models in Monkeys

**DOI:** 10.1371/journal.pone.0040283

**Published:** 2012-07-02

**Authors:** Rossella Falcone, Emiliano Brunamonti, Aldo Genovesio

**Affiliations:** Department of Physiology and Pharmacology, Sapienza University of Rome, Rome, Italy; Cajal Institute, Consejo Superior de Investigaciones Científicas, Spain

## Abstract

We examined whether monkeys can learn by observing a human model, through vicarious learning. Two monkeys observed a human model demonstrating an object–reward association and consuming food found underneath an object. The monkeys observed human models as they solved more than 30 learning problems. For each problem, the human models made a choice between two objects, one of which concealed a piece of apple. In the test phase afterwards, the monkeys made a choice of their own. Learning was apparent from the first trial of the test phase, confirming the ability of monkeys to learn by vicarious observation of human models.

## Introduction

Previous studies have shown that monkeys can learn and acquire motor behaviors by observation. Learning from observation can be very useful for primates, especially when used to learn where to find food and if it is safe and palatable, with the advantage of saving energy and decreasing the risk of harm [1], [2]. However, although several studies have provided evidence that monkeys can learn through the observation of a conspecific [3], [4], [5], [6], the evidence for learning from other primate species, especially humans, remains equivocal.

Research on mirror neurons has shown that monkeys have a shared neural representation of actions performed by human models or monkeys [7], [8], which suggests a neural substrate for learning through the observation of humans. But the ability of monkeys to learn through such observations has been denied [6], [9]. Menieur et al. [6] failed to show any improvement in performance when rhesus monkeys observed a human model performing a concurrent discrimination task and Brosnan and de Waal [9], likewise, reported that capuchin monkeys did not learn to associate tokens with high or low reward values when displayed to them by people.

One interpretation of these negative results is that monkeys might not monitor or pay attention to the humans' behavior as they do with a conspecific. Obviously, the extent to which monkeys attend to human observers and precisely what they attend to will have a crucial effect on what they learn. Flombaum and Santos [10] showed that monkeys are able to attribute significance to human perception, which shows that they attended to the pertinent information in that experiment. Our previous results also required sufficient attention [11]. We used a new nonmatch-to-goal (NMTG) task to test the monitoring abilities of macaque monkeys. This task required monkeys to monitor the human partner's goals. In some trials, after observing a human partner choose one goal, the monkeys were required to switch to a different goal in order to get a reward. We found that monkeys were able to perform this task, showing that in the circumstances of that experiment they could successfully monitor a human's behavior.

A failure in monitoring could explain the previous reports that deny observation learning by monkeys from humans. For example, in the study of Meunier et al. [6], the human models performed an object-discrimination task but did not consume a visible food reward. As a result, the observed events had less salience to a monkey than if food was consumed. The study of Brosnan and de Waal [9], likewise, did not involve the observation of food consumption. Accordingly, we tested whether introducing the vicarious consumption of food by a human model could promote learning in tasks involving the association between objects and rewards. We found that it did.

## Materials and Methods

### Animals

Animal care, housing and experimental procedures conformed to the European (Directive 86/609/ECC) and Italian (D.L. 116/92) laws on the use of nonhuman primates in scientific research. The research protocol was approved by the Italian Health Ministry (Central Direction for the Veterinary Service, approval number 199/2009-B). The housing conditions and the experimental procedures were in accordance with the European law on humane care and use of laboratory animals and complied with the recommendations of the Weatherall report (The use of non-human primates in research).

Both monkeys were monitored daily by the researchers, the animal care staff, and every other day by a veterinarian, to check the general conditions of health and welfare. To enrich their cognitive life, we routinely introduced in the home cage environment toys (often containing items of food that they liked) which promoted their exploratory behavior. Most of the time at the end of each experimental session, the researcher that tested the animals spent additional time interacting with the monkeys directly, giving them, for example, new objects to manipulate. This daily interaction with humans, in addition to the interaction that was part of the task performed, was intended to help the monkeys avoiding potential stress involved in the experiment. To increase the enrichment in the animal housing room, a monitor inside displayed motion pictures.

### Behavioral testing

Two male rhesus monkeys (*Macaca mulatta*), 8.5 Kg and 5.5 Kg, respectively, participated in this study. They were on a controlled diet for the duration of the experiment.

**Figure 1:**
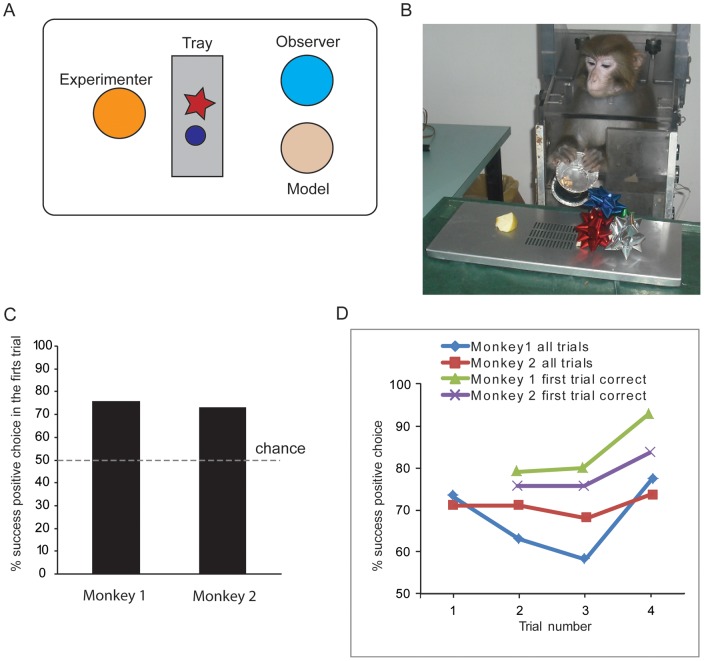
Experimental design and behavioral results. **A**. Testing setup. **B.** The monkey displacing the positive object and exposing the food in the test phase. **C.** Percentage of correct first trials in the test phase. **D**. Performance as a function of trial number during the test phase. Blue and brown lines, all trials for monkey 1 and monkey 2; green and violet lines, 2nd-4th trials after a correct first choice in monkey 1 and monkey 2.

The monkeys lived in a housing room and testing took place in a different room. During a typical testing session, the monkey sat in a primate chair and two humans, the *experimenter* and the *model*, stood nearby. The experimenter stood in front of the monkey, beyond his reach, displaying a metal tray equipped with two different objects. The model stood either on the left or on the right side of the monkey (Fig. 1a). A *problem* consisted of a pair of different objects varying in size, color, and texture–all emotionally neutral. For each pair, only one object in each pair, the positive object, concealed a piece of apple. Over a series of test sessions, spanning ten days, one monkey observed 33 problems and the other 34.

Testing for each pair of objects was divided in two phases: observation and test. In the observation phase, the human experimenter made six presentations of a pair of objects to the human model. These presentations took place within view, but out of reach, of the observer monkey. On all six of these trials, the human model grasped and elevated only the positive object and consumed the piece of apple beneath this object. After each trial, out of the monkey's view, the experimenter placed another piece of apple under the positive object and sometimes changed the position of the positive object, either to the same or to the alternative position on the tray, according to a pseudorandom schedule. The experimenter was careful not to give the monkey any cue as to where the reward was hidden, either by gazing or pointing at the positive object.

In the test phase, the monkey was tested with the same pair of objects used in the just-completed observation phase. The positive object was the same. The test phase consisted of a series of four trials (Fig. 1b). On each trial, the positive object's position was pseudorandomly assigned either to the monkey's left or right, and the monkey was allowed to displace only one of the two objects per trial. After each choice, a new trial followed.

After the test phase had been completed for a given pair of objects, a new pair of objects was used for the next observation phase, followed by another test phase, until the monkey had completed three to five sets of observation–test phases per day.

## Results

### Behavior during the observation phase

Both monkeys showed a similar behavior during the observation phase. They looked at the human model's actions while sitting very quietly at the beginning of a trial, when the model stood in front of the tray. Both monkeys became more active when they saw the model bringing the food to his mouth, occasionally extending their arms toward the human model, as if trying to obtain the food.

### Behavior during the test phase

We calculated the performance of the monkeys on the first trial for all pairs of objects in the test phase. In monkey 1, the positive object in the first trial was presented 18/33 times to the monkey's right and 15/33 to the monkey's left in an unpredictable way. In monkey 2, the positive object was presented 16/34 times in the first trial to the monkey's right and 18/34 to the monkey's left, in a likewise unpredictable sequence.

Both monkeys performed above the chance level of 50% correct. Monkey 1 performed its first choice of 73% correct (24/33) and monkey 2 chose at 71% (24/34) correct. Both scores were significantly different than chance (binomial test, p<0.05) (Fig. 1c).

The first trial can only reflect the effect of observation; later trials could combine this knowledge with learning and exploratory behavior based on the results of the first trial. The performance for the second, third, and fourth trials were, respectively, 63% (20/32), 58% (19/33), 77% (24/31) for monkey 1, and 71% (24/34), 68% (23/34), and 74% (25/34) for monkey 2. Their performance was significantly different than chance (binomial test, p<0.05) in all but the third trials for both monkeys and the second trials for monkey 1.

We also calculated the performance for the second, third and fourth trial, sorting the trials based on the correctness of the first trial. After correct first choices, the percentage of correct choices for the second, third, and fourth trial was 78% (18/23), 79% (19/24), 92% (22/24) in monkey1 and 75% (18/24), 75% (18/24), 83% (20/24) in monkey 2, respectively.

## Discussion

Using an object-reward learning paradigm, we showed that monkeys are able to display vicarious learning from a human model. Previous studies had suggested that monkeys could only learn vicariously from conspecifics [3], [6].

To date, several studies have investigated the ability of monkeys to interact socially with humans. Monkeys can copy some human facial movements, such as lips smacking and tongue protrusion even when they are infants [12], they can be taught to match the experimenter's hand gestures such as clapping hands [13], they can recognize when they are imitated by humans [14] and have an understanding of what humans see [10].

However, a recent study [6] reported that, although monkeys could learn stimulus-reward associations from conspecifics, they failed to learn from a human model. A similar result was found by Brosnan and de Waal [9] in capuchin monkeys, which failed to learn to associate tokens with either high-value or low-value foods by watching the experimenter holding up the token and the corresponding reward. The same monkeys could learn the food's value by watching a monkey model. Brosnan and de Waal [9] have suggested that social attachment and identification with a conspecific might be a requirement for social learning. Furthermore, Brosnan and de Waal [9] interpreted their negative results in support of the hypothesis that only a conspecific can enhance the salience of the objects required to learn. This hypothesis is in line with the identification-based observational learning model that emphasizes the emotional aspects of social learning [9].

However, both in the experiment of Brosnan et al. [9] and that of Meunier et al. [6] monkeys only observed a conspecific consuming the food. They did not watch the human models doing so. By introducing a vicarious reward, we showed that monkeys could learn through the observation of a human model. Therefore, vicarious rewards seem critical in promoting learning, their absence could explain the failures to learn from a human model in previous studies.

Observation learning dates to the Bandura's idea [15] that vicarious rewards enhance imitation because they informs the observer of the consequences of a behavior. Theorists have suggested that it could be important for cooperation and altruism [16]. Among other factors, the acquisition of reward could play the role of positive feedback regarding the success of the behavior [15], [17]. Vicarious reinforcement models support such a contribution, which can be understood as a form of Pavlovian conditioning (stimulus–outcome learning).

V*icarious learning* has been contrasted with *observational learning* [18]. However, our experiment does not bear on that distinction because the monkeys were always rewarded for choosing the positive object, which was learned in the observational phase. Our results shows simply that monkeys can learn object–reward associations by observing these relationships as revealed by human models. In this situation, it seems necessary for the human model to consume the reward and not simply reveal it visually. However, for simpler behaviors, such as copying of left-right foraging choices in a testing apparatus, some learning can be observed even in the absence of a vicarious reward, at least among conspecifics [19].

In our study, as in others [5], [9], [6], the monkeys did not perform perfectly in the test phase. That could be because we offered only six observation trials for each problem. Future experiments should address whether monkeys can reach near-perfect levels of first-trial performance by increasing the amount of time and number of trials dedicated to observation. The improved performance after initial correct trials, in monkey 1, is suggestive of a learning process that continues after the observation phase. The other monkey instead maintained a similar high level of performance in the test trials.

Our study also points to the feasibility of neurophysiological investigations by using human-monkey (H–M) interactive paradigms to investigate the neural correlates of observational learning. Obviously, if the previous negative results were accepted–and monkeys could only learn vicariously from conspecifics–H–M paradigms would be precluded for neurophysiology.

More generally, H–M paradigms can represent a complementary or alternative approach in neurophysiology to the monkey-monkey (M–M) paradigm that has gained prominence in recent years. Several researchers have begun to study social adaptation [20], vicarious rewards [21] and behaviors regarding self vs. others [22] in M–M interaction. The H–M paradigm offers the advantage of a more controlled manipulation of the model's behavior, which becomes an independent variable. The M–M paradigm, by contrast, involves two dependent variables. When adapted to a more controlled experimental setup, H–M paradigms could be adapted to investigate the neural correlates of observational learning at the single-cell level, studied up to now only in neuroimaging experiments [23], [24], [25]. Future studies could asses whether humans could act as models in a variety of learning tasks, such as the conditional motor learning and sequence learning. In conclusion, we have shown that monkeys can learn vicariously from humans and thus opened a the ground for a new line of research that could adopt a human-monkey paradigm for studying social cognition.

## References

[1] Valone TJ, Templeton JJ (2002). Public information for the assessment of quality: a widespread social phenomenon.. Philos Trans.

[2] Snowdon CS, Boe CY (2003). Communication about unpalatable foods in tamarins (Saguinus oedipus).. J Comp Psychol.

[3] Darby CL, Riopelle AJ (1959). Observational learning in the rhesus monkey.. J Comp Physiol Psychol.

[4] Myers WA (1970). Observational learning in monkeys.. J Exp Anal Behav.

[5] Subiaul F, Romansky K, Cantlon JF, Klein T, Terrace H (2007). Cognitive imitation in 2-year-old children (*Homo sapiens*): a comparison with rhesus monkeys (*Macaca mulatta*).. Anim Cogn.

[6] Meunier M, Monfardini E, Boussaoud D (2007). Learning by observation in rhesus monkeys.. Neurobiol Learn Mem.

[7] di Pellegrino G, Fadiga L, Fogassi L, Gallese V, Rizzolatti G (1992). Understanding motor events: a neurophysiological study.. Experimental Brain Research.

[8] Fogassi L, Ferrari PF, Gesierich B, Rozzi S, Chersi F (2005). Parietal lobe: from action organization to intention understanding.. Science.

[9] Brosnan SF, de Waal FB (2004). Socially learned preferences for differentially rewarded tokens in the brown capuchin monkey (Cebus apella).. J Comp Psychol.

[10] Flombaum JI, Santos LR (2005). Rhesus monkeys attribute perceptions to others.. Current Biology.

[11] Falcone R, Brunamonti E, Ferraina S, Genovesio A (2012). Monkeys monitor human goals in a nonmatch-to-goal interactive task.. Plos One.

[12] Ferrari PF, Visalberghi E, Paukner A, Fogassi L, Ruggiero A (2006). Neonatal imitation in rhesus macaques.. PLoS Biol.

[13] Kumashiro M, Ishibashi H, Uchiyama Y, Itakura S, Murata A (2003). Natural imitation induced by joint attention in Japanese monkeys.. Int J Psychophysiol.

[14] Paukner A, Borelli E, Visalberghi E, Anderson JR, Ferrari PF (2005). Macaques (Macaca nemestrina) recognize when they are being imitated.. Biol Lett.

[15] Bandura A (1977). Social learning theory.. Englewood Cliffs, NJ: Prentice Hall.

[16] Fehr E, Fischbacher U (2003). The nature of human altruism.. Nature 425, 785–791.

[17] Miklosi A (1999). The ethological analysis of imitation.. Biol Rev.

[18] Heyes CM, Jaldow E, Dawson G (1993). Observational extinction: observation of non-reinforced responding reduces resistance to extinction in rats.. Anim Learn Behav.

[19] Bonnie KE, de Waal FB (2007). Copying without rewards: socially influenced foraging decisions among brown capuchin monkeys.. Anim Cogn.

[20] Fujii N, Hihara S, Iriki A (2007). Dynamic social adaptation of motion-related neurons in primate parietal cortex.. PLoS One.

[21] Chang SW, Winecoff AA, Platt ML (2011). Vicarious reinforcement in rhesus macaques (*Macaca mulatta*).. Front Neurosci.

[22] Yoshida K, Saito N, Iriki A, Isoda M (2011). Representation of others' action by neurons in monkey medial frontal cortex.. Curr Biol.

[23] Mobbs D, Yu R, Meyer M, Passamonti L, Seymour B (2009). A key role for similarity in vicarious reward.. Science.

[24] Burke CJ, Tobler PN, Baddeley M, Schultz W (2010). Neural mechanisms of observational learning.. Proc Natl Acad Sci U S A.

[25] Monfardini E, Brovelli A, Boussaoud D, Takerkart S, Wicker B (2008). I learned from what you did: Retrieving visuomotor associations learned by observation.. Neuroimage.

